# The relation between dietary intakes and psychological disorders in Iranian adults: a population-based study

**DOI:** 10.1186/s12888-020-02678-x

**Published:** 2020-05-24

**Authors:** Zohreh Sadat Sangsefidi, Masoud Mirzaei, Mahdieh Hosseinzadeh

**Affiliations:** 1 Department of Nutrition, School of Public Health, Shahid Sadoughi University of Medical Sciences, Yazd, Iran; 2 Nutrition and Food Security Research Center, Shahid Sadoughi University of Medical Sciences, Yazd, Iran; 3 Yazd Cardiovascular Research Centre, Shahid Sadoughi University of Medical Sciences, Yazd, Iran

**Keywords:** Psychological disorders, Dietary intakes, Diet

## Abstract

**Background:**

Previous studies showed an association between dietary intakes and psychological disorders. This study aimed to assess the association between dietary intakes and psychiatric disorders in Iran.

**Methods:**

In this cross sectional research, the data on 9965 adults were extracted from enrollment phase of Yazd Health Study (YaHS); a population-based cohort study on Iranian adults which was conducted during 2014 to 2016. Data on socio-demographic characteristics, tobacco use, history of chronic disease, and dietary assessment were collected using a validated researcher-made questionnaire. Moreover, anthropometric measurement was conducted. Psychological and physical activity assessments were also performed by depression, anxiety and stress scale questionnaire (DASS 21 items) and the short form of the International Physical Activity Questionnaire (IPAQ) respectively. Finally, multiple logistic regression analysis was used to evaluate relation between dietary intakes and psychological disorders.

**Results:**

After adjusting for the confounders, egg (depression: OR = 0.72, 95% CI: 0.52–0.98; anxiety: OR = 0.72, CI: 0.55–0.94), fruits (depression: OR = 0.60, 95% CI: 0.43–0.82; anxiety: OR = 0.70, 95% CI: 0.53–0.91), milk (depression: OR = 0.72, CI: 0.58–0.89; anxiety: OR = 0.73, CI: 0.61–0.87), and yogurt (depression: OR = 0.67, CI: 0.47–0.97; anxiety: OR = 0.54, CI: 0.4–0.73) were found to have protective effects on depression and anxiety. Higher fish consumption was associated with greater depression odds (OR = 1.54, CI: 1.18–2.04). Vegetables’ intake had an inverse relationship with anxiety (OR = 0.74, CI = 0.58–0.93) and stress (OR = 0.59, CI: 0.42–0.82). Fruits (OR = 0.6, CI: 0.43–0.85) and milk consumption (OR: 0.61, CI: 0.47–0.77) were found to have protective effects on stress.

**Conclusions:**

Egg, fruits, milk, yogurt, and vegetables’ consumption had an inverse relationship with psychiatric disorders; whereas, higher fish intake was associated with higher depression chance. Further prospective studies are needed to confirm these findings.

## Background

Psychological disorders are not only among the diseases with the highest burden, but also among the most important risk factors of stroke, cardiovascular disease (CVD), and some cancers worldwide [1–5]. Depression, anxiety, and stress are among the most common psychological problems throughout the world [6, 7]. The global prevalence of the major depressive symptoms and anxiety has been estimated as approximately 4.4 and 3.6%, respectively [8]. However, the prevalence rates of anxiety and depression were reported as 21.0 and 20.8% among the Iranian adults, respectively [9].

Environmental factors including diet can influence on psychological health. Some studies evaluated the association between diet and psychological health. Nevertheless, their results are inconsistent [10–31]. For example, some evidences showed a protective effects of consuming fruits [16–19, 21, 30] and vegetables [16–19, 21, 30] on psychological disorders, whereas no significant impact was found in a cross sectional study from Columbia [32]. Legumes [16], dairy products [23–25], and eggs [27] were associated with decreased risk of depression. However, consumption of legumes and dairy products had no significant effect on depression in a prospective population-based study among Taiwanese older people [19]. Some studies reported a preventive role of fish intake against psychological disorders, such as depression [16, 20] and anxiety [28], while no significant relation was found in three cross-sectional data sets (The nationwide Health 2000 Survey, the Fishermen Study and the Finntwin16) [22]. Most previous studies were conducted among the western populations and limited results are available from non-western states, especially the Middle Eastern countries with different dietary intakes and prevalence of psychiatric disorders (e.g. 23.44% in Iran [33] versus 18.5% in the United State [34]). A few large-scale cross sectional studies evaluated the relationship between diet or dietary items and psychological health among the Iranian population [12–15, 35, 36]. These surveys either investigated the general role of diet, as a lifestyle factor, on one psychological disorder [14], or examined the role of dietary patterns [12, 13, 15] and only special dietary items such as eggs [35] or grains [36] on psychological disorders. Only one large-scale cross sectional study specifically evaluated the association between dietary intakes and stress among Iranian adults that reported the protective effect of consuming fruits, vegetables, red meat, and dairy products on stress [31]. However, no population-based study has ever evaluated the relationship between dietary intakes and other psychiatric disorders, such as depression or anxiety among Iranian adults. Since findings of the observational studies on the relation between dietary intakes and risk of psychological disorders are contradictory and little information is available about this issue from the population base studies specially in Iran, this survey was carried out. The aim was to examine the association between dietary intakes and psychological disorders in a large representative sample of Iranian population.

## Materials and methods

### Study population and data collection

We used Yazd Health Study (YaHS) data for the present research. YaHS is a population-based cohort study which has been conducted among a large representative population of Iranian adults (20–69 years old) in Yazd Greater Area. The aim of YaHS was assessing the changing incidence of a variety of chronic disease and associated risk factors in Yazd. Briefly, the recruitment phase of YaHS was performed during September 2014 to March 2016. Adults (*n* = 10,000) from 200 clusters were randomly selected from Yazd population based on residential postal codes using cluster sampling method in 2014. The study was approved by the Ethics Committee of Shahid Sadoughi University of Medical Sciences, Yazd, Iran (Ethical approval code: IR.SSU.REC.1393.7341, Date: July 8, 2014). Furthermore, written informed consents were obtained from all participants. Data on socio-demographic characteristics, tobacco use, history of chronic disease, and dietary assessment were collected using a validated researcher-made questionnaire. In addition, physical activity and psychological health status were evaluated via the valid questionnaires. Anthropometric, measurements were also performed. Reliability and validity of the researcher-made questionnaire was evaluated by consulting experts in each domain of it and a pilot research on 50 participants before performing the study. Reliability of the questionnaire was also confirmed by the Cronbach’s alpha 0.89. More details of YaHS has been recently published elsewhere [37].

### Dietary assessment

Dietary intakes were evaluated using a valid questionnaire [37] by asking about consumptions of fruits and vegetables (serving of consumption per day: Nothing, 1, > 1); milk and yogurt (serving of consumption per week: Nothing, 1–2, > 2), red meat, poultry, egg, legumes (frequency of consumption per week: Never, 1, > 1) and fish (frequency of consumption: Never, < 1 per week, ≥1 per week).

### Anthropometric measurements

Weight was measured using Omron BF511 portable digital scale and body analyzer (Omron Inc. Osaka, Japan) with accuracy of 0.1 kg. Height was measured in a standing position using a tape measure on a straight wall to the nearest centimeter according to standard method. Body Mass Index (BMI) was also computed by dividing the body weight (kg) by the square of height (m).

### Psychological health assessment

The Iranian validated version of depression, anxiety and stress scale questionnaire (DASS 21 items) was used to screen the psychological disorders. DASS 21 is a short version of the self-report depression, anxiety and stress scale questionnaire with seven items per subscale [38]. Respondents read statements about these subscales and record their responses based on a 4-point Likert-type scale ranging from 0 (Did not apply to me at all) to 3 (Applied to me very much or most of the time). For each scale, the scores are summed for identified items. Since the DASS 21 is a short form version of DASS (the Long Form has 42 items), the final score of each scale needs to be multiplied by two (˟2). Finally, depression, anxiety and stress were defined as having the score of ≥10, score of ≥8 and score of ≥15 respectively.

### Physical activity assessment

Assessment of physical activity among participants was conducted using the short form of the International Physical Activity Questionnaire (IPAQ) [39]. The validity of Persian translation of this questionnaire has previously been confirmed by Moghaddam et al. [40]. Finally physical activity levels of subjects were categorized as low, medium and high according to guideline of IPAQ short form [39].

### Statistical analysis

Statistical Package for Social Science (SPSS Inc., Chicago IL. Version 16.0) was applied for analysis. Frequency and percentage were used for description of qualitative variables. Logistic regression analysis was also applied to assess the relation between dietary intakes and psychological disorders in different models with adjustment for various confounders including age (20–29, 30–39, 40–49, 50–59, 60–69 years), education level (secondary school and lower, high school, diploma and Graduate diploma, Bachelors, Masters and PhD), having physical activity (yes/no), history of chronic diseases (yes/no, including hypertension, diabetes, cardiovascular disease, cancer, depression, dyslipidemia), smoking (yes/no) and BMI. Significance level was considered as *p* < 0.05. The lowest frequency or serving of dietary intakes was considered as reference in all models.

## Results

### Participant’s characteristics

The distribution of study population according to general characteristics has been shown in Table 1. According to the results, 50.3% (*n* = 4989) of study population were female and rest of them were men. The participants were predominantly aged 40–49 years (20.7%), married (85%) and secondary school educated or beyond (54.6%). The prevalence of smoking was 10.9%. Moreover, it was found that the prevalence of overweight and obesity among participants was 37.5 and 28.6% respectively. The findings of physical activity assessment also showed that the most of subjects (92.6%) had low (50.8%) or medium (41.8%) physical activity level. Meanwhile, the prevalence of depression, anxiety and stress was estimated 8.6, 13.2 and 6%, respectively.

**Table 1:** General characteristics of study population

Variables	Total (***N*** = 9965) N (%)
**Sex**	
Male	4921 (49.7%)
Female	4989 (50.3%)
**Age (years)**	
20–29	1963 (19.8%)
30–39	2025 (20.4%)
40–49	2049 (20.7%)
50–59	1969 (19.9%)
60–69	1907 (19.2%)
**Education level**	
Secondary school and lower	5389 (54.6%)
Diploma and Graduate diploma	2932 (29.7%)
Bachelors	1291 (13.1%)
Masters and PhD	254 (2.6%)
**Smoking**	
Yes	1056 (10.9%)
No	8610 (89.1%)
**Marital status**	
Married	8430 (85%)
Single	1054 (10.6%)
Widowed	380 (3.8%)
Divorced	55 (0.6%)
**BMI**	
Low weight (< 18.5)	255 (2.7%)
Normal (18.5–24.9)	2944 (31.1%)
Overweight (25–29.9)	3551 (37.5%)
Obesity (≥30)	2707 (28.6%)
**Psychological status**	
**Depression status**	
Normal	8836 (91.4%)
Depression	827 (8.6%)
**Anxiety status**	
Normal	8441 (86.8%)
Anxiety	1279 (13.2%)
**Stress status**	
Normal	9026 (94%)
Stress	1279 (13.2%)
**Physical activity level**	
Low	5059 (50.8%)
Medium	4165 (41.8%)
High	741 (7.4%)

### Description of dietary intakes among participants

Findings about assessing dietary intakes among all participants have been shown in Table 2. The frequency of consumption for red meat, poultry, egg and legumes among most people was more than once per week. Although, fruits intake among most of participants was more than one serving per day, only 23.7% of individuals consumed vegetables more than one serving/day. Moreover, fish consumption once or more than once per week was observed among only 28% of subjects. While, the amount of yogurt intake among most people was more than 2 glasses per week, only 25.2% of subjects drank milk more than 2 glasses.

**Table 2:** Distribution of study population according to dietary intakes

Dietary intakes	Total (***N*** = 9965) N (%)
^**1**^**Red meat**	
Never	263 (2.6%)
Once/week	1651 (16.6%)
> 1/week	8051 (80.8%)
^**1**^**Poultry**	
Never	291 (2.9%)
Once/week	22,923 (29.3%)
> 1/week	6751 (67.7%)
^**1**^**Fish**	
Never	2051 (20.6%)
Lower than once/week	5123 (51.4%)
≥ 1/week	2791 (28%)
^**1**^**Egg**	
Never	733 (7.4%)
Once/week	2923 (29.3%)
> 1/week	6309 (63.3%)
^**1**^**Legumes**	
Never	187 (1.9%)
Once/week	1770 (17.8%)
> 1/week	8008 (80.4%)
^**2**^**Vegetables**	
Nothing	2432 (24.4%)
One/day	5167 (51.9%)
> 1/ day	2366 (23.7%)
^**2**^**Fruits**	
Nothing	917 (9.2%)
One/day	4272 (42.9%)
> 1/ day	4776 (47.9%)
^**2**^**Milk**	
Nothing	3063 (30.7%)
1–2 glasses/week	4393 (44.1%)
> 2 glasses/week	2509 (25.2%)
^**2**^**Yogurt**	
Nothing	520 (5.2%)
1–2 glasses/week	2380 (23.9%)
> 2 glasses/week	7065 (70.9%)

1. Dietary intakes for mentioned items were presented as frequency of consumption

2. Dietary intakes for mentioned items were presented as serving of consumption

### Dietary intakes and depression

The findings of evaluating association between dietary intakes and depression have been presented in the Table 3. After adjustment for several confounders including age, education level, physical activity, chronic diseases history, smoking history and BMI, no significant association was found between consumption of red meat and depression (Once per week: OR: 0.62, CI: 0.37–1.03; More than once per week: OR: 0.74, CI: 0.46–1.19). However, after adjusting for the confounders, fish consumption once or more than once per week was significantly associated with higher depression chance than none-consumption (OR: 1.44, CI: 1.12–1.87). This finding remained unchanged after furthermore adjustment for BMI (OR: 1.54, CI: 1.18–2.04). The persons with intake of egg more than once per week had significantly lower odds of depression in compared with subjects consumed no egg (OR: 0.72, CI: 0.52–0.98). Moreover, individuals who ate fruits more than one serving per day had 37% lower depression chance in comparison to none-consumers (OR: 0.63, CI: 0.46–0.86). This protective impact did not change after more controlling for BMI (OR: 0.6, CI: 0.43–0.82). Similarly, drinking 1–2 glasses of milk per week was meaningfully related to decreased depression chance than none-consumption (OR: 0.72, CI: 0.58–0.89). This association remained unchanged after furthermore adjustment for BMI (OR: 0.72, CI: 0.58–0.89). In addition, it was observed that subjects who consumed yogurt more than 2 glasses per week had significantly lower odds of depression than none-consumers (OR: 0.68, CI: 0.47–0.97). An additional adjustment for BMI did not change this finding (OR: 0.67, CI: 0.47–0.97). However, there was no significant relation between other dietary intakes and depression.

**Table 3:** Multivariable-adjusted odds ratios (OR) (95% CI) for depression across different frequencies or servings for dietary intakes in study population (*N* = 9965)

Dietary intakes	^**3**^Depression			
	^**4**^Multivariable adjusted		^**5**^Multivariable + BMI	
	OR	CI	OR	CI
^**1**^**Red meat**				
Never	Reference		Reference	
Once/week	^*^0.6	^*^0.37–0.98	0.62	0.37–1.03
> 1/week	0.69	0.44–1.09	0.74	0.46–1.19
^**1**^**Poultry**				
Never	Reference		Reference	
Once/week	0.95	0.55–1.64	0.93	0.54–1.61
> 1/week	0.99	0.58–1.67	0.96	0.56–1.63
^**1**^**Fish**				
Never	Reference		Reference	
Lower than once/week	0.89	0.7–1.13	0.93	0.73–1.19
≥ 1/week	^*^1.44	^*^1.12–1.87	^*^1.54	^*^1.18–2.01
^**1**^**Egg**				
Never	Reference		Reference	
Once/week	0.8	0.58–1.1	0.81	0.59–1.12
> 1/week	0.73	0.54–1.004	^*^0.72	^*^0.52–0.98
^**1**^**Legume**				
Never	Reference		Reference	
Once/week	1.37	0.76–2.45	1.38	0.76–2.52
> 1/week	1.11	0.62–1.96	1.16	0.64–2.09
^**2**^**Vegetables**				
Nothing	Reference		Reference	
One/day	0.92	0.74–1.15	0.94	0.74–1.18
> 1/ day	0.8	0.6–1.07	0.83	0.62–1.11
^**2**^**Fruits**				
Nothing	Reference		Reference	
One/day	0.88	0.66–1.18	0.83	0.61–1.11
> 1/ day	^*^0.63	^*^0.46–0.86	^*^0.6	^*^0.43–0.82
^**2**^**Milk**				
Nothing	Reference		Reference	
1–2 glasses/week	^*^0.72	^*^0.58–0.89	^*^0.72	^*^0.58–0.89
> 2 glasses/week	0.99	0.78–1.26	1.03	0.81–1.31
^**2**^**Yogurt**				
Nothing	Reference		Reference	
1–2 glasses/week	1.00	0.69–1.44	0.96	0.66–1.4
> 2 glasses/week	^*^0.68	^*^0.47–0.97	^*^0.67	^*^0.46–0.97

^1^ Dietary intakes for mentioned items were presented as frequency of consumption

^2^ Dietary intakes for mentioned items were presented as serving of consumption

^3^depression was defined as having the score of ≥10 from the DASS21 questionnaire

^4^Adjusted for age (20–29, 30–39, 40–49, 50–59, 60–69 years), education level (Secondary school and lower, High school, Diploma and Graduate diploma, Bachelors, Masters and PhD), physical activity level (low, medium, high), history of chronic diseases (hypertension, diabetes, cardiovascular disease, cancer, depression, dyslipidemia), smoking (yes/no)

^5^Adjusted for age (20–29, 30–39, 40–49, 50–59, 60–69 years), education level (Secondary school and lower, High school, Diploma and Graduate diploma, Bachelors, Masters and PhD), physical activity level (low, medium, high), history of chronic diseases (hypertension, diabetes, cardiovascular disease, cancer, depression, dyslipidemia), smoking (yes/no) and BMI. ^*^Significance level was considered as *p* < 0.05

### Dietary intakes and anxiety

Table 4 shows the findings about association between dietary intakes and anxiety. Those who used egg more than once per week had meaningfully lower anxiety chance than none-consumers (OR: 0.75, CI: 0.58–0.98). This relation remained unchanged after more controlling for BMI (OR: 0.72, CI: 0.55–0.94). Furthermore, the persons with consumption of vegetables more than one serving per day had 26% lower anxiety chance in compared with those consumed no vegetables (OR: 0.74, CI: 0.58–0.93). This protective effect remained after furthermore adjustment for BMI (OR: 0.74, CI: 0.58–0.93). Similarly, the chance of anxiety among individuals who used fruits more than once serving per day was significantly lower than none-consumers (OR: 0.70, CI: 0.54–0.90). This finding did not change after more controlling for BMI (OR: 0.70, CI: 0.53–0.91). Moreover, drinking1–2 glasses of milk per week was meaningfully associated with decreased odds of anxiety than none-consumption (OR: 0.71, CI: 0.6–0.85). This association remained after furthermore adjustment for BMI (OR: 0.73, CI: 0.61–0.87). Meanwhile, anxiety chance among subjects with consumption 1–2 glasses of yogurt was significantly lower than none-consumers (OR: 0.55, CI: 0.41–0.74). An additional adjustment for BMI did not change this relation (OR: 0.54, CI: 0.4–0.73). But, no significant association was observed between other dietary intakes and anxiety.

**Table 4:** Multivariable-adjusted odds ratios (OR) (95% CI) for anxiety across different frequencies or servings for dietary intakes in study population (*N* = 9965)

Dietary intakes	^**3**^Anxiety			
	^**4**^Multivariable adjusted		^**5**^Multivariable + BMI	
	OR	CI	OR	CI
^**1**^**Red meat**				
Never	Reference		Reference	
Once/week	1.25	0.79–1.98	1.46	0.9–2.39
> 1/week	1.23	0. 8–1.91	1.49	0.93–2.39
^**1**^**Poultry**				
Never	Reference		Reference	
Once/week	1.05	0.66–1.66	1.11	0.69–1.79
> 1/week	1.21	0.77–1.89	1.27	0.8–2.03
^**1**^**Fish**				
Never	Reference		Reference	
Lower than once/week	0.83	0.69–1.01	0.82	0.67–1.00
≥ 1/week	1.12	0.91–1.38	1.12	0.9–1.39
^**1**^**Egg**				
Never	Reference		Reference	
Once/week	0.84	0.64–1.1	0.82	0.62–1.07
> 1/week	^*^0.75	^*^0.58–0.97	^*^0.72	^*^0.55–0.94
^**1**^**Legume**				
Never	Reference		Reference	
Once/week	1.2	0.73–1.97	1.16	0.69–1.95
> 1/week	1.01	0.62–1.65	0.98	0.59–1.62
^**2**^**Vegetables**				
Nothing	Reference		Reference	
One/day	0.92	0.77–1.1	0.93	0.77–1.12
> 1/ day	^*^0.74	^*^0.58–0.93	^*^0.77	^*^0.6–0.98
^**2**^**Fruits**				
Nothing	Reference		Reference	
One/day	0.81	0.64–1.03	0.79	0.61–1.01
> 1/ day	^*^0.7	^*^0.54–0.9	^*^0.7	^*^0.53–0.91
^**2**^**Milk**				
Nothing	Reference		Reference	
1–2 glasses/week	^*^0.71	^*^0.6–0.85	^*^0.73	^*^0.61–0.87
> 2 glasses/week	0.97	0.79–1.18	0.98	0.8–1.2
^**2**^**Yogurt**				
Nothing	Reference		Reference	
1–2 glasses/week	0.75	0.56–1.02	^*^0.72	^*^0.52–0.98
> 2 glasses/week	^*^0.55	^*^0.41–0.74	^*^0.54	^*^0.4–0.73

^1^ Dietary intakes for mentioned items were presented as frequency of consumption

^2^ Dietary intakes for mentioned items were presented as serving of consumption

^3^Anxiety was defined as having the score of ≥8 from the DASS21 questionnaire

^4^Adjusted for age (20–29, 30–39, 40–49, 50–59, 60–69 years), education level (Secondary school and lower, High school, Diploma and Graduate diploma, Bachelors, Masters and PhD), physical activity level (low, medium, high), history of chronic diseases (hypertension, diabetes, cardiovascular disease, cancer, depression, dyslipidemia), smoking (yes/no)

^5^ Adjusted for age (20–29, 30–39, 40–49, 50–59, 60–69 years), education level (Secondary school and lower, High school, Diploma and Graduate diploma, Bachelors, Masters and PhD), physical activity level (low, medium, high), history of chronic diseases (hypertension, diabetes, cardiovascular disease, cancer, depression, dyslipidemia), smoking (yes/no) and BMI

^*^Significance level was considered as *p* < 0.05

### Dietary intakes and stress

The findings of assessing the relation between dietary intakes and stress had been shown in Table 5. Vegetables consumption was inversely associated with stress (OR: 0.59, CI: 0.42–0.82). This protective effect remained significant after more controlling for BMI (OR: 0.59, CI: 0.42–0.82). Similarly, fruits intake was associated with lower stress chance (OR: 0.59, CI: 0.42–0.83). This association did not change after further adjustment for BMI (OR: 0.6, CI: 0.43–0.85). It was also observed that the subjects who drank 1–2 glasses of milk per week had decreased odds of stress than none-consumers (0.60: 0.47–0.76). An additional adjustment for BMI did not change this finding (0.61: 0.47–0.77). However, no association was found between other dietary intakes and stress.

**Table 5:** Multivariable-adjusted odds ratios (OR) (95% CI) for stress across different frequencies or servings for dietary intakes in study population (*N* = 9965)

Dietary intakes	^**3**^Stress			
	^**4**^Multivariable adjusted		^**5**^Multivariable + BMI	
	OR	CI	OR	CI
^**1**^**Red meat**				
Never	Reference		Reference	
Once/week	0.87	0.48–1.55	0.9	0.5–1.73
> 1/week	0.96	0.55–1.66	1.08	0.6–1.94
^**1**^**Poultry**				
Never	Reference		Reference	
Once/week	1.41	0.73–2.74	1.37	0.7–2.68
> 1/week	1.31	0.68–2.51	1.27	0.66–2.44
^**1**^**Fish**				
Never	Reference		Reference	
Lower than once/week	0.79	0.61–1.02	0.77	0.59–1.01
≥ 1/week	1.2	0.91–1.6?	1.22	0.91–1.63
^**1**^**Egg**				
Never	Reference		Reference	
Once/week	0.75	0.53–1.07	0.77	0.54–1.11
> 1/week	0.75	0.54–1.06	0.75	0.53–1.07
^**1**^**Legume**				
Never	Reference		Reference	
Once/week	1.38	0.67–2.82	1.31	0.63–2.7
> 1/week	1.24	0.61–2.5	1.17	0.58–2.38
^**2**^**Vegetables**				
Nothing	Reference		Reference	
One/day	^*^0.72	^*^0.56–0.92	^*^0.71	^*^0.55–0.91
> 1/ day	^*^0.59	^*^0.42–0.82	^*^0.59	^*^0.42–0.82
^**2**^**Fruits**				
Nothing	Reference		Reference	
One/day	^*^0.68	^*^0.5–0.92	^*^0.68	^*^0.5–0.94
> 1/ day	^*^0.59	^*^0.43–0.83	^*^0.6	^*^0.43–0.85
^**2**^**Milk**				
Nothing	Reference		Reference	
1–2 glasses/week	^*^0.6	^*^0.47–0.76	^*^0.61	^*^0.47–0.77
> 2 glasses/week	0.81	0.62–1.06	0.77	0.58–1.03
^**2**^**Yogurt**				
Nothing	Reference		Reference	
1–2 glasses/week	0.94	0.62–1.43	0.85	0.56–1.3
> 2 glasses/week	0.82	0.54–1.22	0.74	0.49–1.12

^1^Dietary intakes for mentioned items were presented as frequency of consumption

^2^ Dietary intakes for mentioned items were presented as serving of consumption

^3^Stress was defined as having the score of ≥15, score of ≥10 and score of ≥8from the DASS21 questionnaire

^4^Adjusted for age (20–29, 30–39, 40–49, 50–59, 60–69 years), education level (Secondary school and lower, High school, Diploma and Graduate diploma, Bachelors, Masters and PhD), physical activity level (low, medium, high), history of chronic diseases (hypertension, diabetes, cardiovascular disease, cancer, depression, dyslipidemia), smoking (yes/no)

^5^Adjusted for age (20–29, 30–39, 40–49, 50–59, 60–69 years), education level (Secondary school and lower, High school, Diploma and Graduate diploma, Bachelors, Masters and PhD), physical activity level (low, medium, high), history of chronic diseases (hypertension, diabetes, cardiovascular disease, cancer, depression, dyslipidemia), smoking (yes/no) and BMI

^*^Significance level was considered as *p* < 0.05

## Discussion

The present research indicated an inverse relationship between intakes of eggs, fruits, milk, and yogurt with depression and anxiety. More consumption of vegetables might be associated with decreased odds of anxiety and stress, whereas higher consumption of fish might be related to greater chance of depression. Furthermore, an inverse association was found between intakes of fruits and milk with stress.

Similar to our results, a protective effect was reported for fruits and vegetables consumption against stress among the university students in European countries [41], the United Kingdom [21], China [30], as well as Iran [31]. Fruits’ intake was also associated with lower depression risk in the European countries [41] and United Kingdom [21].

In contrast to our survey, one research from Columbia showed no significant association between fruits’ consumption and depression among a sample of overweight and obese women [32]. The validated short form of the Center for Epidemiological Studies Depression Scale was used for psychological health assessment in the Columbian study; while we applied the Iranian validated version of DASS 21 in this research. The differences between our results and some studies can be explained by discrepancies in the population’s characteristics, dietary habits, and the applied psychological health assessment tools.

The protective effects of vegetables and fruits might be attributed to nutrients, such as carbohydrates, vitamins, antioxidants, and phytochemicals [5]. The possible association of carbohydrates contained in such foods with psychological health may be due to the secretion of insulin caused by consumption of these foods, which consequently stimulated the production of neurotransmitters such as serotonin by the entry of tryptophan into the brain [42–44]. Furthermore, the nutrients in vegetables and fruits, including folate and vitamin B6 [45], antioxidants (such as vitamins C, E) [18], phytochemicals (such as polyphenols) [18, 46], and minerals (such as iron, calcium and magnesium) [47] could have a protective role against the psychological problems.

The findings of the present paper are in line with the surveys from Iran [31] and Japan [23] that reported a preventive effect for intake of the dairy products on stress and depression, respectively.

Contrary to our research, no relationship was observed between the dairy products consumption and stress or depression among the university students in a survey by El Ansari et al. [21]. In the mentioned study, the Cohen’s Perceived Stress Scale and modified Beck Depression Inventory were applied for examining the participants’ psychological health; whereas, we used the Iranian validated version of DASS 21 in our study. The various characteristics of population, dietary habits, and different tools for assessing the psychiatric disorders in the mentioned study [21] might explain the reason of this discrepancy.

The protective role of dairy products can be related to their minerals such as calcium, because calcium, as a neurotransmitter, is critical in the function of neuromuscular tissues involved in the emotional regulation and consequently prevents from the psychological disorders [48]. Dairy products might also have a protective effect due to their anti-inflammatory process [23], since they contain compounds such as calcium [49], lactoferrin [50], and bioactive peptides [51].

In contrast to our results, a preventive role was found for fish consumption against stress and depression in the study by El Ansari et al. [21] as well as against anxiety and depression in a research by Jacka et al. [28]. The protective impact of fish intake was attributed to its omega-3 fatty acids due to having anti-inflammatory activity in the previous studies [52, 53]. However, fish consumption was generally low among participants of our study. Fried fish was also used by most of the consumers. It was also found that fried foods’ consumption [54] and diet rich in fat [55] were related to increased depression risk caused by the production of free radicals and promotion of pro-inflammatory states [55, 56] that follows consumption of fried fish. Furthermore, many surveys have indicated that frying fish might lead to loss of the omega three fatty acids content in fish [57–60]. Therefore, the relationship observed between higher fish consumption and higher depression chance in our study might be explained by consumption of fried fish.

A few researches [61] evaluated association between egg intake and psychological disorders. Shafiei et al. (2017) [61] found no significant relation between egg consumption and psychiatric disorders. Evidence has shown that the nutrients including tryptophan [62], B vitamins [10, 27, 45, 63], vitamin D [64], and omega-3 fatty acids [65] can have a beneficial impact on psychological health and prevents from the psychiatric disorders. Therefore, egg consumption might be associated with decreased odds of psychological problems, because egg contains the mentioned nutrients [35].

The current survey enjoys from several strengths. To the best of our knowledge, it is the first population-based study that presents the relationship between several dietary intakes and a variety of psychological disorders including depression, anxiety, and stress within a large population in a Middle Eastern country. Moreover, we controlled for an extensive range of confounding factors that might influence the individuals’ psychological status. However, our study had some limitations. First, as a cross-sectional study, it may not accurately explain the causality among the study variables. Thus, a cohort and controlled dietary study is needed to establish causality. The measurement error, as an identified feature of any dietary assessment method, was another limitation of this research. We also could not control the effect of all confounders due to the unknown or unmeasured factors. Since foods are consumed together, a single food may not completely explain the etiology of chronic disease such psychological disorders. Therefore, dietary pattern approach in future population-based studies can prepare a comprehensive picture of foods and their interactions and consider the combined effects of them.

## Conclusions

The present study showed an inverse association between consuming egg, fruits, milk, and yogurt with depression and anxiety. In addition, more consumption of vegetables might be related to decreased odds of anxiety and stress. An inverse association was observed between higher intake of fruits and milk with stress, whereas higher consumption of fish might be related to higher odds of depression. Further studies, especially population-based cohort research, are suggested to provide more conclusive evidence to explain the association between dietary intakes and psychological disorders.

## Data Availability

The datasets used and analyzed during the present study are available from the corresponding author on reasonable request.

## Abbreviations

YaHS: Yazd health study
OR: Odds ratio
CI: Confidence interval
CVD: Cardiovascular disease
(DASS 21 items): Depression, anxiety and stress scale questionnaire
BMI: Body mass index
IPAQ: International physical activity questionnaire
